# PD-1/PD-L1 and DNA Damage Response in Cancer

**DOI:** 10.3390/cells12040530

**Published:** 2023-02-07

**Authors:** Mateusz Kciuk, Damian Kołat, Żaneta Kałuzińska-Kołat, Mateusz Gawrysiak, Rafał Drozda, Ismail Celik, Renata Kontek

**Affiliations:** 1 Department of Molecular Biotechnology and Genetics, University of Lodz, Banacha 12/16, 90-237 Lodz, Poland; 2 Doctoral School of Exact and Natural Sciences, University of Lodz, Banacha Street 12/16, 90-237 Lodz, Poland; 3 Department of Experimental Surgery, Faculty of Medicine, Medical University of Lodz, Narutowicza 60, 90-136 Lodz, Poland; 4 Department of Immunology and Allergy, Medical University of Lodz, Pomorska 251, 92-213 Lodz, Poland; 5 Department of Gastrointestinal Endoscopy, Wl. Bieganski Hospital, 91-347 Lodz, Poland; 6 Department of Pharmaceutical Chemistry, Faculty of Pharmacy, Erciyes University, 38039 Kayseri, Turkey

**Keywords:** cytotoxic drugs, DNA damage response, immunotherapy, programmed death ligand-1 (PD-L1)

## Abstract

The application of immunotherapy for cancer treatment is rapidly becoming more widespread. Immunotherapeutic agents are frequently combined with various types of treatments to obtain a more durable antitumor clinical response in patients who have developed resistance to monotherapy. Chemotherapeutic drugs that induce DNA damage and trigger DNA damage response (DDR) frequently induce an increase in the expression of the programmed death ligand-1 (PD-L1) that can be employed by cancer cells to avoid immune surveillance. PD-L1 exposed on cancer cells can in turn be targeted to re-establish the immune-reactive tumor microenvironment, which ultimately increases the tumor’s susceptibility to combined therapies. Here we review the recent advances in how the DDR regulates PD-L1 expression and point out the effect of etoposide, irinotecan, and platinum compounds on the anti-tumor immune response.

## 1. Introduction

Surgery, chemotherapy, and radiation therapy constitute the main approaches to cancer treatment. One alternative way of targeting cancer concentrates on boosting the patient’s immune system so that it can act against the tumor. The term “cancer immunotherapy” refers to a set of treatment methods that involve either the modification of the immune system of the host or the employment of immune system components to combat cancer. Cytokines, immune cells, and monoclonal antibodies are a few examples of immune system components that have been utilized in the treatment of cancer [1]. Due to the fact that cancer cells are derived from normal cells, they may not express antigens against which an immune response can be developed [2]. 

Although there are several cancer antigens distinguished, they may not trigger a significant level of the immune response. Moreover, the antigen may not be efficiently uptaken, processed, and presented, leading to a diminished and insufficient immunological response. Afterwards, activation of specific T and B cells in the lymph nodes is necessary for the immune cells to migrate to the tumor site, where they can act. In case of the successful elimination of tumor cells, the second wave of antigen presentation may occur and the phenomenon known as “epitope spreading” could expand the immune repertoire that is directed against the tumor [2].

The death of cancer cells in response to the drug may be sufficient to trigger an immune response [3]. The release of antigens can activate the T-cells [4] that detect the dying cancer cells exhibiting calreticulin (CRT) and phosphatidylserine (PS) on their surface. These molecules are recognized by the immune cells such as macrophages enabling the elimination of cancer cells [5,6]. Immature dendritic cells (DCs) and macrophages express the transmembrane receptor CD91 (also known as LRP1) on their surface that interacts with CRT on tumor cells. Moreover, the autophagy-dependent release of ATP work as a recognition signal for DCs exhibiting P2X purinoceptor 7 (P2RX7) on the surface and leads to the inflammasome-mediated secretion of interleukin 1β (IL-1β). Moreover, the secretion of high-mobility group protein 1 (HMGB1)—a ligand for toll-like receptor (TLR-4)—is required for DCs activation and effective antigen presentation [7]. These molecules, called damage-associated molecular pattern molecules (DAMPS), stimulate the activation of the immune system [8,9].

**Figure 1:**
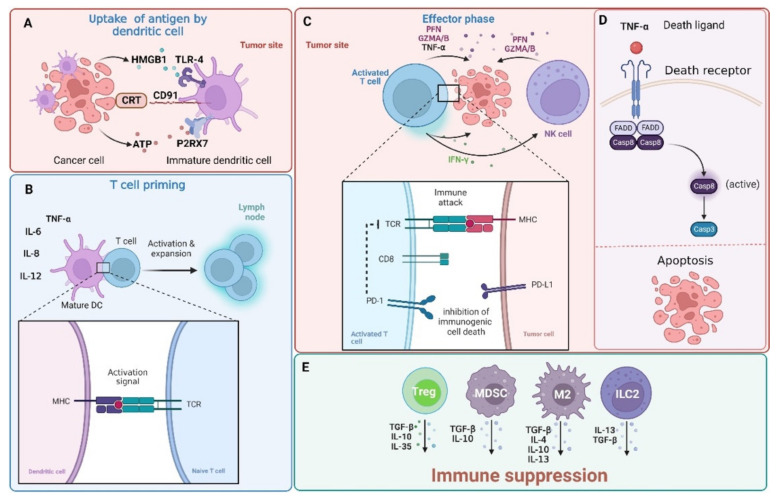
The interaction between tumor cells and immune cells. Tumor cells are detected by adaptive (comprising of B and T cells) and innate immune system (mainly natural killer (NK) cells). Effector CD4+ and cytotoxic CD8+ T cells are capable of recognizing peptide antigens presented on MHC class II or MHC class I, respectively. (**A**) Immunogenic cell death (ICD) is associated with the release of damage-associated molecular patterns (DAMPs) from dying cells. Such molecular patterns, whether expressed on the surface of cells or released outside of the cell, can promote tumor antigen presentation and boost adaptive immunity. Calreticulin (CRT) expressed on cancer cells interacts with the CD91 receptor on dendritic cells (DCs) and high-mobility group protein 1 (HMGB1)—a ligand for the toll-like receptor (TLR-4) receptor on DCs is released by cancer cells. Additionally, ATP released from cancer cells interacts with the P2X purinoceptor 7 (P2RX7) on DSc. (**B**) DCs are required for cytotoxic CD8+ T cell priming. In this process, DCs uptake antigens from tumor cells undergoing apoptosis and with the support of CD4+ helper cells present them to CD8+ T cells. Moreover, DCs can secrete tumor necrosis factor α (TNF-α,) and interleukins (IL-6, IL-8, and IL-12) that help to trigger the anticancer immune response. (**C**) Activated CD8+ T cells can kill cancer cells through the release of granzyme A/B (GZMA/B), perforins (PFN), or interferon gamma (INFγ) and TNFα that (**D**) induce cell death through the activation of the extrinsic apoptosis pathway. The extrinsic pathway is triggered with the binding of certain ligands to the TNFα receptor super family. This leads to the oligomerization of the receptor and activation of procaspase-8 through the recruitment of adaptor proteins and the formation of a death-inducing signaling complex (DISC). (**E**) Cancer cells reshape the tumor immune microenvironment into an immunosuppressive surrounding that is rich in regulatory T cells (Tregs), myeloid-derived suppressor cells (MDSCs), group 2 innate lymphoid cells (ILC2s), and tumor-associated macrophages (M2) that release immunosuppressive interleukins (IL-4, IL-10, IL-13, and IL-35) or transforming growth factor β (TGF-β) and up-regulate receptors (such as programmed cell death 1 ligand 1 (PD-L1)) that “hide” tumor cells from the immune recognition (not shown here). This topic has been extensively reviewed in [15,16,17] and will not be discussed in detail here. Casp—caspase; FADD—FAS-associated death domain protein; TCR—T-cell receptor. Created with BioRender.com accessed on 1 February 2023.

All these components work in concert to bring about the sequential events of tumor cell recognition and recruitment of DCs, phagocytosis, antigen processing, maturation, and presentation to T lymphocytes. Last but not least, the cascade produces an interferon (IFN)-mediated immune response involving γδ T cells and cytotoxic CD8+ T lymphocytes (Figure 1) [10].

As described above, the immune response can identify cancer cells and eliminate them through a variety of processes that involve cooperation between the innate and adaptive branches of the immune system. T-cells play a significant part in this process. The activation of these cells helps to activate an immune response that attacks cancer cells. A specific peptide epitope of the antigen has to be displayed on the major histocompatibility complex (MHC) of an antigen-presenting cell (APC), and it has to form a complex with the T-cell receptor on T cells to become activated. The second signal resulting from the interaction of co-stimulatory molecules is essential for T-cell activation. T-cells will enter the unresponsive condition of clonal anergy if they are not given any co-stimulatory molecules to work with [11]. There are examples of both positive and negative regulators of this process. While positive regulators boost anti-tumor activity, negative regulators impede the killing process and instead promote the growth of tumors. Therefore, an immunotherapy that targets the negative regulators to increase anti-tumor responses could be a viable alternative therapeutic method of treating cancer [12].

Immune checkpoint pathways, such as the programmed death receptor-1 and programmed death ligand-1 (PD-1/PD-L1) signaling pathway, are crucial for the regulation of immune self-tolerance that can be employed by cancer cells to avoid immune surveillance. Inhibition of these T cell surface receptors leads to elevated autoimmunity, which in turn generates an immune response against cancer [13,14]. 

Monoclonal antibodies (mAbs) targeting PD-1/PD-L1 immune checkpoint have led to a remarkable anti-tumor response in many cancer types [18]. There are now three anti-PD-1 immune checkpoint inhibitors that are licensed for use in clinical settings. These inhibitors include pembrolizumab [19,20,21,22], nivolumab [23,24,25,26], and cemiplimab [27,28]. Additionally, approval was given for three anti-PD-L1 antibodies: atezolizumab [29,30,31], avelumab [32,33,34], and durvalumab [35,36,37]. The targeting of PD-1/PD-L1 with mAbs has been reviewed by many authors [12,38,39].

## 2. PD-1/PD-L1 Pathway

PD-1, also known as CD279, is a transmembrane protein anchored in the cell membrane of activated T and B lymphocytes. PD-1 plays a crucial role in preventing immune-mediated tissue damage by suppressing the actions of self-reactive and inflammatory effector T lymphocytes against non-hematopoietic tissues [40,41]. The protein is built up of an extracellular IgV domain (ED), a transmembrane domain (TM), and an intracellular cytoplasmic tail (ICT). ICT comprises tyrosine-bearing signaling motifs: immunoreceptor tyrosine-based switch motif (ITSM) and immunoreceptor tyrosine-based inhibitory motif (ITIM) [42,43]. Phosphorylation of ITIMs and ITSMs leads to the recruitment of Src homology region 2 domain-containing phosphatases (SHP-1 and SHP-2) [43,44]. SHP2 is preferably recruited to the ITSM motifs, while SHP1 can bind both tyrosine motifs [45]. This event suppresses PI3K/AKT/mTOR [46] and PLCg-1/RAS/MEK/ERK1/2 [47] (for full names of the proteins see the Abbreviations section) signaling pathways responsible for the control of interleukin 2 (IL-2) expression and leads to the degradation of forkhead box protein O1 (FOXO1)—a transcription factor capable of PD-1 expression control. This results in the down-regulation of PD-1 levels [48,49]. SHP-1 and SHP-2 inhibit subsequent T-cell receptor (TCR) signaling pathways exhibiting the negative effect of T cell proliferation and the generation of cytokines including IFN-γ and IL-2, which eventually results in immune evasion by tumor cells [11]. Evidence shows that at least one of the mechanisms by which PD-1 inhibits activation of the PI3K/AKT pathway relies on phosphatidylinositol 3,4,5-trisphosphate 3-phosphatase and the dual-specificity protein phosphatase/casein kinase II (PTEN/CK2) axis [50]. This topic was recently reviewed in [43].

PD-1 binds to particular ligands known as programmed cell death ligands PD-L1 (B7-H1) and PD-L2 (B7-DC) that belong to glycoproteins present on the surface of tumor cells. PD-L2 is thought to be expressed only in APCs, including DCs, macrophages, monocytes, and some B cells. Limited evidence suggests it may also be involved in tumor evasion in some cases [51,52,53].

In the PD-L1 structure, ED, TM, and the intracytoplasmic region can be distinguished. The ED domain of PD-L1 encompasses Ig variable distal and proximal regions. The intracytoplasmic region contains three conserved amino acid motifs: RMLDVEKC, DTSSK, and a QFEET motif. The RMLDVEKC motif is crucial for the phosphorylation of the signal transducer and activator of transcription 3 (STAT3) and the other DTSSK motif works as a suppressor of this process [54,55]. Binding of PD-L1 to PD-1 results in a reduction in the generation of cytokines as well as a suppression of the proliferation and function of T lymphocytes [11,12,54]. The expression of PD-L1 in cancer cells is controlled by several signaling pathways and proteins often mutated or up-regulated during malignant transformation, including COX2/mPGES1/PGE2 [56], hypoxia-inducible factor alpha (HIF1α) [57,58,59], nuclear factor NF-kappa-B p105 subunit (NF-κB) [60], PI3K/AKT/mTOR [61,62], RAF/MEK/ERK/MAPK [63,64,65,66] pathway, and STATs [67,68].

## 3. Canonical DNA Damage Response

DNA damage response (DDR) plays a central role in the control of the genomic stability of cells. DNA damaging agents trigger complex signaling pathways that direct the fate of damaged cells. Depending on the type of insult, different pathways are triggered to finally provide output reliant on the abundance of the trigger and the metabolic state of the cell. These signaling events may result in (a) damage tolerance, (b) DNA repair, (c) cell death, or (d) cell-cycle arrest. Despite the large number of DNA damage types that can be introduced in the DNA structure by a variety of DNA-damaging factors, the DDR signaling shares common features [69]. Here we focus on the cellular response to the DNA strand breaks as it is the prevailing type of DNA damage introduced by anti-cancer agents.

The DDR pathway is always initiated with the detection of damage by specific proteins. Double-strand breaks (DSBs) are sensed by MRN complexes composed of MRE11-RAD50-NBS1 proteins. This event leads to the recruitment and activation of serine-protein kinases, including ataxia telangiectasia mutated (ATM). Furthermore, the mediator of DNA damage checkpoint protein 1 (MDC-1) is recruited at the damage sites and works as a platform for ATM recruitment, leading to its retention at the chromatin close to the damage location. ATM undergoes autophosphorylation and works as a master regulator of response to DSBs as it phosphorylates a multitude of targets in the DDR including MDC1, nibrin (NBS1), TP53-binding protein 1 (53BP1), breast cancer type 1 susceptibility protein (BRCA1), and histone protein H2AX. The phosphorylation of H2AX leads to the formation of γH2AX foci (H2AX protein phosphorylated on Ser139) that recruits the MDC-1 platform protein and provides a positive feedback loop that amplifies the damage response. MDC-1 also supports the formation of repair complexes through the recruitment of E3 ubiquitin-protein ligases including RNF4, RNF8, and RNF168 that are necessary for the recruitment of other factors, including 53BP1 and BRCA1 to the sites of damage [70,71,72]. 

In contrast, single-stranded DNA (ssDNA) that occurs at stalled replication forks as a result of minichromosome maintenance (MCM2-7) complex helicase activity and as an intermediate in DNA repair pathways is sensed by serine/threonine-protein kinase ATR. Similar to ATM, ATR kinase exists as an inactive dimer that undergoes autophosphorylation and dissociation in response to DNA damage. ATR is recruited to the replication protein A/ATR-interacting protein (RPA-ATRIP) complexes formed on the ssDNA. Moreover, full activation requires the presence of other factors including topoisomerase 2-binding protein 1 (TOPBP1) and a cell cycle checkpoint control protein complex 9-1-1 (composed of RAD9-RAD1-HUS1) [69,71].

In the downstream events, ATR and ATM kinases activate serine/threonine-protein kinases CHK1 and CHK2 responsible for the inhibition and degradation of M-phase inducer phosphatases CDC25A and CDC25C, which normally contribute to the progression of the cell cycle through the dephosphorylation of cyclin–cyclin-dependent kinase (CDK) complexes. Furthermore, the activation of cellular tumor antigen p53 (TP53) protein up-regulates the expression of pro-apoptotic proteins and cell cycle inhibitors that ultimately results in cell cycle arrest or cell death [71,73,74]. This topic has been reviewed by multiple authors and will not be discussed here in detail [75,76,77].

## 4. DNA Strand Break Repair

Poly [ADP-ribose] polymerase 1 (PARP-1) is one of the earliest responders to strand breaks that catalyze the transfer of polymer ADP-ribose residues (PAR) to many downstream targets including histones, DNA repair proteins, transcription factors, as well as PARP-1 itself. This event allows the relaxation of DNA molecules near the damage site and recruitment of other DDR proteins containing PAR-binding modules that are involved in multiple pathways of DNA repair, including base excision repair (BER) and nucleotide excision repair (NER), mismatch repair (MMR), single-strand break (SSBR), and double-strand break (DSBR) repair proteins [70,72,78].

DSBs can be repaired in one of two major ways: homologous recombination (HR) [79] or non-homologous end joining (NHEJ) [80]. The pathway that is used to repair DSBs is influenced by the phase of the cell cycle and the status of the chromatin. PARP-1 can detect breaks in the DNA, and its activity plays a role in the early recruitment of various proteins such as damage sensors MRE11 and NBS1. The early recruitment of MRE11 nuclease may also promote DNA repair via HR. Moreover, the interaction of ATM with PARs can stimulate kinase activity in vitro. PARP1 is also essential for the early and prompt mobilization of BRCA1 to DSBs. In contrast, PARP1 may restrict the activity of HR through PAR-ylation of BRCA1 allowing its association with receptor-associated protein 80 (RAP80) to inhibit this type of DNA repair [78].

HR repair represents a high-fidelity mechanism of DSB repair. It is initialized with the recruitment of MRE11 that exhibits endo- and exo-nucleolytic activity that allows the formation of ssDNA fragments. Moreover, it interacts with CtBP-interacting protein (CtlP) and BRCA1 which help to control the resection process. Furthermore, CDKs and ATM phosphorylate CtBP-interacting protein (CtIP) which promotes end resection and HR through the recruitment of DNA helicase BLM and exonuclease 1 (EXO1) to the sites of DNA damage. In the next step, ssDNA is covered with RPA protein followed by recruitment of platform protein RAD52 and RAD51 recombinase that controls DNA strand invasion together with BRCA2 protein [71,72,79,81].

In contrast, the NHEJ pathway is activated in response to the DSBs that arise throughout the cell cycle. It is initiated with the binding of X-ray repair cross-complementing protein 5/6 (KU70/80) and DNA-dependent protein kinase catalytic subunit (DNA-PKcs) that form nucleoprotein complexes often described as synapses. Other NHEJ components include X-ray repair cross-complementing protein 4 (XRCC4), non-homologous end-joining factor 1 (NHEJ1/XLF), and DNA ligase IV that form complexes and allow completion of the repair process. Similarly to HR, the NHEJ repair pathway is controlled by PARP-1. PARP-1 binds and stimulates the catalytic activity of DNA-PKcs. Moreover, it may promote the recruitment of chromatin modeling enzymes to sites of DSBs for efficient repair [71,82,83]. 

## 5. DNA Damage Response and PD-1/PD-L1

Radiotherapy and chemotherapy induce DNA damage in cancer cells, which ultimately lead to their elimination. Recent research has provided evidence to support the hypothesis that DDR is a significant element impacting the effectiveness of cancer immunotherapy. The effect of conventional chemotherapy on the immune system is beginning to emerge. This is due to the fact that such agents are usually tested for their anticancer activity in cell cultures in vitro and immunodeficient mice and do not include any immunological follow-ups [84]. Accumulating evidence suggests that (re)activation of tumor-targeted immune responses is essential to the success of both conventional and targeted anticancer treatments (in addition to their direct cytostatic/cytotoxic effects). Through the enhancement of the immunogenicity of malignant cells and blockage of the immunosuppressive systems developed by cancers, the combination of chemotherapy with immune checkpoint inhibitors can provide significant benefits for cancer patients [84]. 

For example, it was found that exposure of cancer cells to physical DNA damaging factors including ionizing radiation [85,86] and chemical factors that induce DNA damage such as topoisomerase inhibitors (camptothecin [87], doxorubicin [88,89,90], and irinotecan [91]), alkylating agents (carboplatin [92,93], cisplatin [94,95,96,97,98], oxaliplatin [99], and mitomycin C [100]), or antimetabolites such as decitabine [101,102,103] and 5-fluorouracil (5-FU) [104,105,106] can lead to up-regulation of PD-L1 expression in the treated cells. Moreover, a growing body of evidence indicates that the effectiveness of PD-1/PD-L1 therapy may be connected to genomic instability (particularly microsatellite instability (MSI)) [107,108,109,110,111,112,113]. This profound response of tumors exhibiting high genomic instability may be partially explained by excessive production and release of peptide neoantigens that favor the recruitment or activation of tumor-infiltrating lymphocytes (TILs) and result in up-regulation of PD-1/PD-L1 in immune and cancer cells [114]. The up-regulation of PD-L1 in cancer cells might also increase the availability of epitopes to which anti-PD-L1 agents can bind and might further explain the therapeutic efficacy of the combined treatments. Indeed, tumors expressing high levels of PD-L1 respond well to anti-PD-1/PD-L1 treatments [115,116]. Multiple studies have shown that increased expression of PD-L1 also occurs following CDK2/4/6 [117,118] and serine/threonine-protein kinase mTOR [119] inhibition and that targeting of the above-mentioned kinases and simultaneous treatment with anti-PD-1/PD-L1 agents may enhance the therapeutic effect exerted by these agents alone.

The up-regulation of PD-L1 expression in response to DSBs-inducing agents such as etoposide and camptothecin or ionizing radiation is dependent on the ATM/ATR/CHK1 pathway. Moreover, the cells that survive the exposure to ionizing radiation revert to normal expression levels of PD-L1, indicating only transient up-regulation of this immune checkpoint molecule in response to DNA damage. Moreover, cells lacking crucial DSBR pathway components including BRCA2 or Ku70/80 exhibit up-regulated levels of PD-L1, and this enhanced expression of the protein is mainly due to CHK1 activation following DNA end resection by EXO1 rather than the result of DSB occurrence. This was confirmed with an experiment using the CHK1 inhibitor in BRCA2-defective cells, where up-regulation of PD-L1 was not observed. Furthermore, PD-L1 up-regulation in the absence of KU-80 and DNA damage is probably due to the build-up of replication-associated DSBs to which KU80 may bind. In summary, tumors defective in BRCA2 or KU70/80 exhibit higher expression of PD-L1 following ionizing radiation treatment due to the excessive rates of DNA end resection in the absence of KU70/80 that promotes ATR-CHK1 signaling and PD-L1 up-regulation [100]. 

Furthermore, in reaction to DSBs, both STAT1/3 and interferon regulatory factor 1 (IRF1) become active. It is important to note that the overexpression of PD-L1 requires IRF1, which suggests that the DSB-dependent up-regulation of PD-L1 is driven by the classical JAK1/2-STAT1/2/3-IRF1 pathway [100,106,120,121,122]. This is consistent with the early findings on the mechanism of INFγ-induced up-regulation of PD-L1 [123]. 

## 6. Cytotoxic Agents, DDR, and Immunotherapy 

### 6.1. Irinotecan

Irinotecan is one of the best-studied topoisomerase I inhibitors. It has been employed in the treatment of various types of malignancies such as pulmonary, pancreatic, gastric, ovarian, cervical, and colorectal cancer. Irinotecan prevents the relegation of strand breaks introduced during the physiological activity of the enzyme through covalent binding with topoisomerase. The formed ternary complex composed of the drug, DNA, and topoisomerases work as an obstacle for replication and transcription machinery. Its collision with the machinery results in the formation of DSBs and activation of the ATM-CHK2-TP53 signaling pathway which eventually leads to DNA repair or apoptosis, depending on the severity and abundance of damage [124].

**Figure 2:**
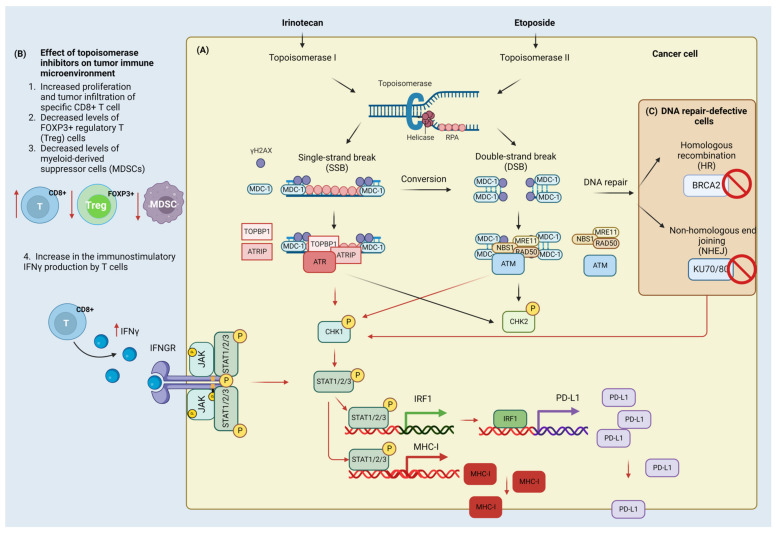
Effect of irinotecan and etoposide on the DNA damage (DDR) and immune response. Cancer cell (**A**) Irinotecan inhibits DNA topoisomerase I which leads to the formation of single-strand breaks (SSBs) and double-strand breaks (DSBs). Single-stranded DNA (ssDNA) in the replication fork that arises due to helicase activity is coated with replication protein A (RPA) protein and a mediator of DNA damage checkpoint protein 1 (MDC-1). MDC-1 serves as a platform protein for topoisomerase 2-binding protein 1 (TOPBP1), ATR-interacting protein (ATRIP), and serine/threonine-protein kinase (ATR). In contrast, DSBs that arise through the conversion of SSBs and activity of topoisomerase II inhibitors (here etoposide) are detected by the MRN complex composed of double-strand break repair protein MRE11 (MRE11), nibrin (NBS1), and DNA repair protein (RAD50). The MRN complex is involved in the recruitment and activation of ATM kinase. Activated ATR and ATM phosphorylate have multiple targets including checkpoint kinases (CHK1/2). The phosphorylation of H2AX and accumulation of MDC-1 contribute to further recruitment of ATR/ATM kinases and amplification of damage signaling. CHK1 activation leads to the phosphorylation of signal transducers and activator of transcription (STAT) proteins that control (up-regulate) the expression of interferon regulatory factor (IRF1) and the major histocompatibility complex class I molecule (MHC-I). IRF-1 works as a transcription factor for programmed death ligand-1 (PD-L1) protein. Tumor immune microenvironment (**B**) Topoisomerase inhibitors stimulate the anti-tumor immune response through the increased proliferation and infiltration of CD8+ cells and the production of immunostimulatory interferon gamma (INFγ). INFγ binds to and activates the interferon-gamma receptor (INFGR) that contributes to the activation of tyrosine-protein kinase JAK (JAK)/STAT signaling to confer the increased PD-L1 expression. Moreover, it also reduces the regulatory FOXP3+ T-cells and myeloid-derived suppressor cells (MDSC) that normally attenuate the anti-tumor immune response. Cells defective in double-strand break repair components (**C**): Inactivation of the breast cancer type 2 susceptibility protein (BRCA2) of homologous recombination (HR) pathway and X-ray repair cross-complementing protein 5/6 (KU70/80) of the non-homologous end joining (NHEJ) pathway contributes to the activation of CHK1/STAT/IRF/PD-L1 signaling. Created with BioRender.com accessed on 1 February 2023.

According to the findings of McKenzie et al., patient-derived melanoma cell lines are more vulnerable to T-cell-mediated cytotoxicity after being treated with topoisomerase I inhibitors, such as topotecan, camptothecin, and irinotecan. This effect is highly dependent on the TP53 mutational status. The T-cell response is highly proficient when the TP53 is wild-type or possesses a mutation that does not affect the TP53 activity [125]. Irinotecan treatment in murine models of cancer have provided evidence that the drug may affect the tumor immune environment in several ways, including (a) increased proliferation of tumor-specific CD8+ T cell, (b) increase in immunostimulatory IFNγ production, (c) decreased amount of FOXP3+ regulatory T cells [91], and (d) myeloid-derived suppressor cells (MDSCs) that are normally responsible for immunosuppressive effects with (e) an accompanying overall increase in MHC class I and PD-L1 expression in tumor cells. Moreover, irinotecan exhibited a synergic effect when given anti-PD-L1 antibodies [84,91,126]. In 2015, the liposomal version of irinotecan known as Nal-IRI received approval from regulatory authorities around the world for the treatment of metastatic pancreatic adenocarcinoma in conjunction with 5-FU. It has also been demonstrated that Nal-IRI can increase the T-cell-mediated cytotoxicity that is directed toward tumor cells in vivo. As a result, when paired with an anti-PD-1 mAb, it exhibited a greatly improved anticancer activity. This combined treatment increased the infiltration of CD8+ T lymphocytes into the tumor, capable of producing cytotoxic effects [124,125,127,128]. The impact of irinotecan on the DDR and the tumor microenvironment is shown in Figure 2.

### 6.2. Etoposide

About two-thirds of small-cell lung cancer (SCLC) patients have the advanced-stage disease at diagnosis that is associated with a poor prognosis and a 5-year survival rate of 7%. This is attributed to the high rates of tumor cell proliferation, metastasis, and resistance to currently available therapeutic options. The tumor is characterized by a high tumor mutational burden (TMB) in many genes, which suggests that DNA errors brought on by cell replication and mutations continue to build up and the immune system is unable to recognize and destroy cancer cells [129]. For more than 20 years, the gold standard for SCLC has been chemotherapy with platinum (either carboplatin or cisplatin) combined with etoposide. In conjunction with carboplatin and etoposide, atezolizumab was the first immune checkpoint inhibitor to be introduced as a first-line therapeutic option for extensive-stage small-cell lung cancer (ES-SCLC) [130]. Recent studies have shown that immune checkpoint inhibitors can improve the prognosis of patients with ES-SCLC [131,132]. 

Etoposide (epipodophyllotoxin) is another example of topoisomerase II poisons that introduce DSBs [133]. As a consequence of DSBs occurrence, the ATM signaling pathway is triggered which in turn results in the activation of CHK2 kinase [134,135]. A hypersensitivity to etoposide and an increased rate of chromosomal abnormalities constitute the consequences of mutations in the ATM kinase, which occurs in cells derived from patients with ataxia telangiectasia [136,137]. The activation of ATM in reaction to etoposide treatment entails the generation of the MRN complex foci [138,139,140]. Etoposide simultaneously activates ATR-mediated pathway and contributes to the recruitment of the 9–1–1 complex and activation of CHK1 kinase due to the accumulation of ssDNA coated with RPA protein [134,135,141,142]. The presence of these RPA foci following etoposide treatment may reflect an attempt by the cells to repair DSBs introduced by the agent through the HR pathway [143]. Moreover, inhibition of DNA replication relies heavily on ATR activation [135]. 

The majority of etoposide-induced topoisomerase-II-mediated DNA damage can be repaired by NHEJ. This is consistent with the results of Malik et al. that have established the key role of yeast KU70 of the NHEJ pathway for the survival of Saccharomyces cerevisiae upon etoposide treatment [144,145,146]. Additionally, cells deficient in KU70 and KU80 but not those with defective DNA-PKcs exhibit sensitivity to the etoposide treatment [147]. In contrast, Palmitelli et al. suggest that DNA-PKcs inhibition significantly improves DNA damaging effects of etoposide with a twofold higher rate of chromatid breaks and exchanges [148]. However, HR seems to be the primary pathway of etoposide-induced damage repair. The formation of strand breaks associated with etoposide treatment is associated with a reduction in the levels of KU70, KU86, and DNA-PKcs in addition to an increase in the concentration of RAD51 protein, suggesting HR-mediated repair [149,150].

Cancer stem cells (CSCs) are a subpopulation of cells that have both the ability to self-renew and the capability to develop into other cell types. It was discovered that cancer cells can go through the epithelial-to-mesenchymal transition (EMT) in response to stimuli from the cells that are present in the microenvironment of the tumor [151]. In turn, this results in the production of cells that have characteristics that are comparable with those of CSCs. In addition, the release of IL-6 into the milieu of the tumor by CSCs is the primary factor responsible for the transformation of non-stem cancer cells into CSCs [152,153]. Additionally, IL-6 induces the expression of other cytokines that are advantageous for the formation of CSCs. This topic was recently reviewed by our group [154]. Elevated expression of PD-L1 as a consequence of the EMT/β-catenin/STAT3 pathway activation improves the immune evasion of CSCs. Etoposide was shown to suppress the EMT/β-catenin/STAT3 pathway and down-regulate PD-L1 expression in cancer cells [155]. It has been demonstrated that etoposide can stimulate tumor-specific immunity, in which CD8+ cytotoxic T cells play an important role [156,157,158]. 

Contrasting evidence comes from the in vitro studies of etoposide treatment in breast cancer cells and bone marrow stromal cells, where the drug induces up-regulation of PD-L1 on the cancer cell surface [159,160]. Moreover, teniposide (etoposide derivative) induces DNA damage in tumor cells, which is associated with the activation of the NF-κB pathway and stimulation of interferon genes protein (STING)-dependent type I interferon signaling. Both of these pathways contribute to the activation of DCs, which leads to the activation of T cells. In addition, teniposide enhances the anticancer activity of anti-PD1 treatment in a variety of mouse tumor models [161]. The DDR induction by etoposide and its influence on the tumor microenvironment is presented in Figure 2.

### 6.3. Cisplatin and Other Platinum Compounds

Many types of human solid tumors are successfully treated with chemotherapeutic drugs that crosslink DNA [162]. Two types of DNA crosslinks can be distinguished. The intrastrand crosslink covalently connects two nucleotides of the same DNA molecule, while the interstrand crosslinks (ICLs) develop when two different DNA strands are linked. These latter crosslinks require bifunctional alkylating agents that have two reactive groups that can form covalent bonds with DNA molecules [163]. ICLs constitute one of the most difficult-to-repair lesions that cells encounter during their lifetime. The formation of ICLs triggers DDR in cancer cells. The repair is carried out with the cooperative aid of at least five repair pathways i.e., the Fanconi anemia (FA) pathway, HR, NER, MMR, and translesion DNA synthesis (TLS) [164,165]. Unrepaired ICLs prevent strand separation of DNA during DNA replication and transcription. As a consequence, cells die from mitotic catastrophe or apoptosis [166]. 

Cisplatin has been widely used in the treatment of many cancer types. However, the fundamental restrictions of cisplatin’s therapeutic use are the side effects and resistance mechanisms that commonly accompany cisplatin-based therapy. Soon after the introduction of cisplatin into the clinic, other platinum compounds were developed to mitigate the negative effects of cisplatin. These include two of the most comprehensively studied analogs of the drug: oxaliplatin and carboplatin [10,167].

Despite the belief that platinum-based treatments lack cell cycle selectivity, cytotoxicity is boosted when the drug is delivered to cells during the S phase of the cell cycle. Cisplatin induces a G2 cell cycle arrest and subsequent accumulation of cells in the G2/M phase through the inhibition of the CDK2-cyclin A or B kinase, which is followed by apoptosis [168]. Only a minuscule fraction of the intracellular platinum can form a complex with the DNA. Platinum compounds can interact with RNA [169,170] or various proteins which may confer the platinum-based compound’s side effects and toxicity [171,172]. Although physically different, cisplatin and oxaliplatin produce similar adducts at analogous locations in the DNA molecule [173]. The cisplatin-induced DNA damage is detected by more than 20 different proteins from different DNA repair pathways including hMSH2 or hMUTSa (MMR), nonhistone chromosomal high-mobility group 1 and 2 (HMG1/2) proteins, the human RNA polymerase I transcription ‘upstream binding factor’ (hUBF), and the transcriptional factor ‘TATA-binding protein’ (TBP). Therefore, it is plausible to postulate that DNA damage triggers many seemingly unrelated biological consequences, each of which can be initiated by a different recognition protein [174].

**Figure 3:**
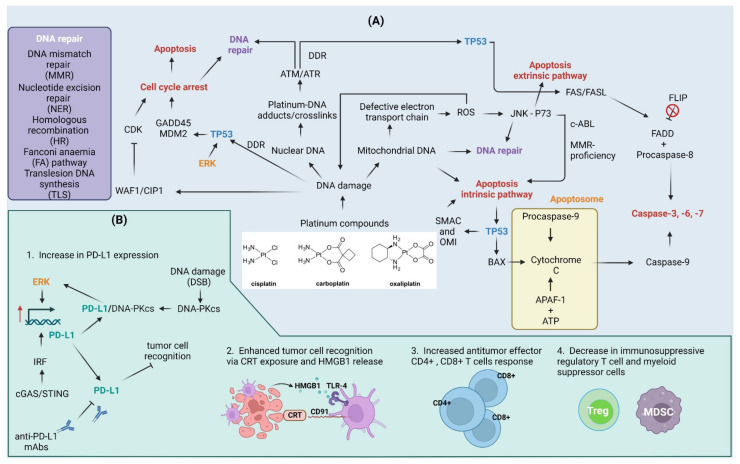
Cellular and immune response to platinum compounds. (**A**) Cellular response to platinum compounds: Platinum compounds induce DNA damage to both nuclear and mitochondrial DNA. Mitochondrial DNA damage can lead to defects in the electron transport chain that contribute to electron leakage and the formation of reactive oxygen species (ROS). ROS contribute to DNA damage in both nuclear and mitochondrial DNA in a feedback mechanism. ROS also act as molecular messengers and contribute to the activation of the Jun amino-terminal kinase (JNK) pathway that together with the P73 protein trigger apoptotic cell death. C-ABL kinase activity and mismatch repair (MMR)-proficiency are required for cell death to occur. DNA damage activates DNA damage response (DDR), as indicated by the activation of serine/threonine kinases ATM and ATR. The outcome of DDR relies heavily on cellular tumor antigen p53 (TP53) protein: (a) activation of TP53 in response to DNA damage triggers cell cycle arrest through up-regulation of cyclin-dependent kinase inhibitor 1 (WAF1/CIP1), 45kd-growth arrest, and DNA damage (GADD45) and mouse double minute 2 homolog (MDM2) or via WAF1/CIP1-mediated inhibition of cyclin-dependent kinases (CDKs); (b) subsequent DNA repair via co-operation of nucleotide excision repair (NER), MMR, homologous recombination (HR), Fanconi anemia (FA) pathway, or translesion synthesis (TLS); or (c) apoptosis induction through intrinsic apoptosis pathway followed by ROS-damage accumulation, TP53-dependent up-regulation of Bcl2-associated agonist of cell death (BAD), Diablo IAP-binding mitochondrial protein (SMAC) and serine protease HTRA2 (OMI), or through activation of FASL/FAS extrinsic apoptosis pathway. Cisplatin induces flice-like inhibitory protein (FLIP) ubiquitination and degradation, which further activate the extrinsic pathway. (**B**) Effect of platinum compounds on the antitumor immune response: Cyclic GMP-AMP synthase (cGAS)/stimulator of interferon genes protein (STING) pathway and extracellular signal-regulated kinase (ERK) signaling up-regulate programmed death ligand-1 (PD-L1) expression. The DNA-dependent protein kinase catalytic subunit (DNA-PKcs) up-regulated in response to double-strand breaks (DSBs) associates with PD-L1 conferring to the activation of the ERK pathway and further up-regulation of PD-L1. This, on the one hand, contributes to immune evasion, but also increase the therapeutic efficiency of anti-PD-L1 monoclonal antibodies (mAbs). Platinum compounds also enhance tumor recognition through calreticulin (CRT) exposure on cell surface and high mobility group protein 1 (HMGB1) release, induce CD4+/CD8+ T cell response, and suppress regulatory T cells (Treg) and myeloid-derived suppressor cells (MDSC). APAF-1—apoptotic protease-activating factor 1; FADD—FAS-associated death domain protein. Created with BioRender.com accessed on 1 February 2023.

Multiple molecular mechanisms of action were proposed for platinum compounds such as cisplatin (Figure 3). For example, cisplatin was found to induce oxidative stress through the excessive generation of reactive oxygen species (ROS). The concentration of cisplatin and the length of exposure time are two of the most important factors that determine the generation of ROS in cancer cells [175]. ROS cause both nuclear (nDNA) and mitochondrial (mtDNA) DNA damage at rates of approximately 104 DNA lesions per cell of an organism every day. These lesions include modified bases, interstrand and DNA-protein crosslinks, SSBs, and DSBs [176,177]. The Jun amino-terminal kinase (JNK) signaling plays a crucial role in cellular responses to oxidative stress. JNK triggers several responses, including DNA repair, antioxidant synthesis, and cell death, depending on the strength and length of the damage signal [178]. P73 is a homolog of the TP53 protein that is involved in cell cycle regulation and apoptosis. Following treatment of cells with cisplatin and transplatin, P73 establishes a complex with JNK leading to apoptotic cell death [179]. Two factors are necessary for cisplatin to induce P73-dependent apoptosis: ABL tyrosine kinase activation and the cellular proficiency of the MMR repair pathway that connects damage sensing to apoptotic signaling [174].

ATM, ATR, and DNA-PKcs are all rapidly activated in response to oxidative DNA damage [180,181]. Nuclear foci composed of ATR and H2AX are formed at the location of DNA damage in cells treated with cisplatin. ATR blockade using a dominant-negative mutant prevents cisplatin-induced TP53 activation and renal cell death. In ATR-deficient fibroblasts, cisplatin-induced TP53 activation and apoptosis are attenuated. Both CHK1 and CHK2 are phosphorylated in response to cisplatin treatment; however, CHK1 phosphorylation leads to the proteasomal degradation of the kinase. Therefore, ATR-CHK2 activation plays a crucial role in TP53-mediated apoptosis response [182,183]. In U87-MG glioma cells, cisplatin treatment results in apoptosis with concomitant up-regulation of cyclin-dependent kinase inhibitor 1 (WAF1/CIP1) which works as a suppressor of CDKs activity [184]. Cisplatin causes apoptosis of ovarian cancer cells by increasing the levels of TP53 in mitochondria and inducing the release of Diablo IAP-binding mitochondrial protein (SMAC), cytochrome c, and serine protease HTRA2 (OMI). Apoptosis mediated by cisplatin is dependent on TP53 and necessitates the release of SMAC. The release of SMAC is directly triggered by TP53 in mitochondria [185]. There is also evidence that TP53 can directly bind to cisplatin-damaged DNA complexes [167,186]. 

The apoptotic response triggered in response to platinum compounds is highly dependent on TP53 phosphorylation by a multitude of activated signaling pathways including extracellular signal-regulated kinase (ERK) [187,188], RAC-alpha serine/threonine-protein kinase (AKT) [189], and protein kinase C (PKC) [190]. ERK phosphorylates TP53 leading to up-regulation of WAF1/CIP1, 45kD-growth arrest and DNA damage (GADD45), and mouse double minute 2 homolog (MDM2), providing the time necessary to repair induced DNA damage [191]. Notably, phosphorylation of Bcl2-associated agonist of cell death (BAD) on Ser-112 is mediated through the ERK pathway, while Ser-136 phosphorylation is dependent on the PI3K/AKT pathway. Moreover, inhibition of the above-mentioned pathways sensitizes cancer cells to cisplatin [189]. Similarly, inhibition of AKT results in enhanced accumulation of TP53 in mitochondria and the release of SMAC conferring the greater efficacy of cisplatin [185]. Furthermore, cisplatin also induces the extrinsic apoptosis pathway as indicated by the activation of the FAS receptor, caspase 8, and effector caspases 3, -6, and -7 activation [192]. Moreover, cisplatin triggers apoptosis and flice-like inhibitory protein (FLIP) ubiquitination and degradation in a TP53-dependent manner in vitro [193].

Platinum compounds exhibit a variety of effects on the anti-cancer immune response (Figure 3). The up-regulation of PD-L1 in response to platinum compounds [9,93] is potentially induced through the activation of the ERK1/2 pathway in vitro and in vivo [97,98]. Moreover, PD-L1 associates with DNA-PKcs that contribute to the activation of the ERK pathway and as a feedback mechanism for the up-regulation of PD-L1. The inhibition of PD-L1 with monoclonal antibody (H1A) sensitizes human triple-negative breast cancer cells to cisplatin in vitro and in vivo [99], suggesting that the combination of the drug with anti-PD-L1 treatments may enhance the therapeutic efficacy of chemotherapeutic drug alone [9,194,195], at least in mouse tumor models. Moreover, cisplatin and oxaliplatin may have different effects on CD8+ T cell fraction and function. Oxaliplatin seems to act as a stronger activator of CD8+ T cells than cisplatin in preclinical models of head and neck cancer [9]. Moreover, oxaliplatin was found to promote PERK/eIF2α/caspase 8/BAP31 signaling (see abbreviations section) to facilitate CRT exposure on the cancer cell surface and HMGB1 release in a mouse model of colorectal cancer [196].

As mentioned above, the tumor microenvironment can be shifted from an immunosuppressive to an immune-supportive state following the use of platinum compounds. This is accompanied by the activation of dendritic cells (CD80+ and CD86+), increased antitumor effector CD4+, CD8+ T cells response, and decreased immunosuppressive regulatory T and myeloid suppressor cells ratios as observed in mice models of ovarian, lung, and colon cancer [92,194,195]. An increase in CRT, MHC class I, antigen presentation, and T-cell infiltration are some of the mechanisms by which cisplatin improves tumor immunogenicity after both short- and long-term exposure. The cyclic GMP-AMP synthase (cGAS)/STING pathway is triggered in response to cisplatin treatment. With the increased expression of PD-L1, MHC-I, and CRT in tumor cells, cGAS/STING overexpression changes tumor immunogenicity and improves anti-PD-L1 treatments in in vitro and in vivo mice models [95,197]. Conflicting results were obtained in clinical studies of platinum-based chemotherapy on PD-L1 expression, where the treatment resulted in a decrease in the immune checkpoint molecule in tumors from lung cancer patients [198]. However, both the additive and synergic effects of platinum compounds and anti-PD-L1 antibodies have been described in the literature [94,95,197,199].

## 7. Current Challenges and Future Prospects

**Figure 4:**
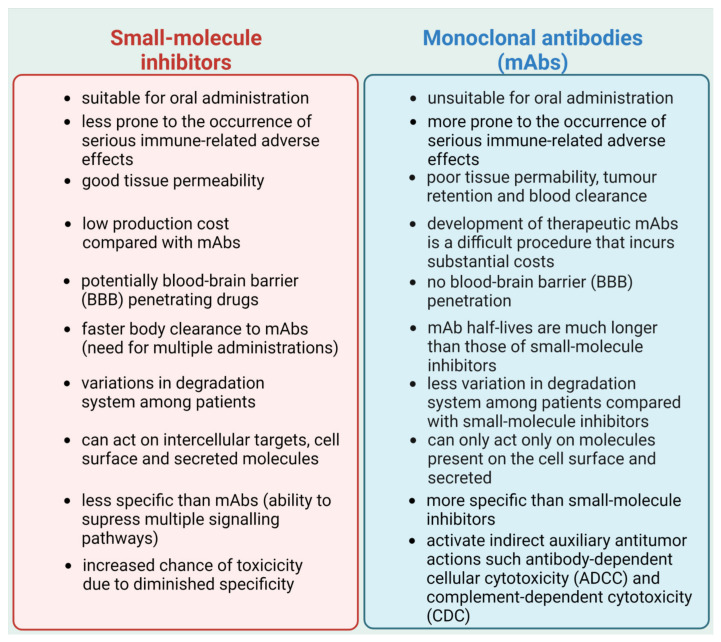
Comparison of small-molecule inhibitors and monoclonal antibodies (mAbs). Based on [202]. Created with BioRender.com accessed on 1 February 2023.

The field of immunotherapy is constantly expanding. Immune checkpoint inhibitors are a promising new treatment option for cancer treatment. Multiple clinical trials have shown that immunotherapy can extend both progression-free survival (PFS) and overall survival (OS) in cancer patients. The abundance of mAbs that have been granted FDA approval and the number of cancer types for which they are used continues to grow, which is reflective of the enthusiasm for PD-1/PD-L1 inhibition and its potential application in clinical practice [200]. Still, mAbs possess many drawbacks, such as a lack of oral bioavailability or poor permeability of tumor tissues, which leads to the overall low response rate of PD-1/PD-L1 inhibition that limits their clinical effectiveness. Additionally, mAbs therapy may drive immunogenicity issues and cause serious immune-related adverse effects (irAEs), with possible deadly outcomes [201,202,203]. Alternatively, many small molecule inhibitors may have certain advantages in dealing with these problems (Figure 4). Small molecule inhibitors are more suitable for oral administration and are less prone to the occurrence of serious irAEs. Moreover, they are less expensive, have better tissue permeability, and possess many more other important characteristics, which make them more favorable for potential clinical use compared with mAbs [202].

Many crystal structures were published after the FDA approved the use of monoclonal antibodies targeting the PD-1/PD-L1 axis, demonstrating the interaction mechanisms of antibodies as well as small-molecule compounds with PD-1 and PD-L1. These studies provided information on hot spots that can be employed for the design of new, more specific compounds targeting this pathway [11]. Moreover, they showed that the PD-1/PD-L1 protein interaction surface is large and hydrophobic, with no profound binding pocket making it difficult to target with small-molecule inhibitors. The hydrophobic nature of this hot spot also necessitates the use of hydrophobic molecules with significant adverse properties including toxicity and low water solubility [204]. Nonetheless, multiple compounds from different chemical classes, including biphenyls [205,206,207], terphenyls [208,209], biphenyls with a ring fusion [210], elongated biphenyls [211], and symmetric biphenyls [212,213], were tested for anti-PD-1/PD-L1 interaction; however, they relied heavily on binding assay without their examination in biological systems. Moreover, the number of false-positive hits is still too high as in the case of salicylates NCI 211717 and NCI 211845. Moreover, when screening drugs with in vivo mouse models, structural variation between mouse and human PD-1/PD-L1 should be considered as it can greatly affect druggability profiles [204]. Humanized knock-in animals could help to circumvent this obstacle [214].

Currently, immunotherapy is used with different forms of treatment to obtain a long-lasting antitumor clinical response in patients who are resistant to monotherapy. In many cases, PD-L1 is up-regulated in response to chemotherapeutic agents. Besides their cytotoxic effect, chemotherapeutic drugs may enhance the tumor infiltration of CD8+ T cells and NK cells, the maturation of APCs (DCs or tumor macrophages), and in some circumstances, the activity of MDSCs as evidenced by animal model studies. In this manner, primary cytostatic and cytotoxic drugs re-establish an immune-reactive tumor microenvironment, which increases the tumor’s sensitivity to PD-L1-targeted monoclonal antibodies. However, the challenge is to combine the chemotherapeutic drugs with anti-PD-L1 agents to surpass the effectiveness of agents alone and alleviate the side effects of the drugs. A given cytotoxic drug or regimen needs to be selected not only based on its ability to effectively kill cancer and inhibit the expansion of cancer cells but also based on its tendency to modulate the activity of immune-active cells and to sustain the activity of immunotherapy. Moreover, many currently used drugs have not been examined in the context of immune activation against the tumor cells or their utility in combinations with checkpoint inhibitors [8]. 

However, one of the most significant drawbacks of chemotherapy is still a lack of specificity. Chemotherapy will target both the highly proliferating tumor cells as well as normal cells including lymphocytes. Therefore, lymphopenia is one of the most common side effects associated with the use of DNA-damaging agents. This can hamper the utility of immune–chemotherapy drug combinations for the treatment of cancer. This limitation could be overcome with the use of targeted agents, which exhibit lower toxic effects for normal cells compared with chemotherapy. However, the effect of such agents and their combinations, not only on PD-L1 expression but also on the alterations in the tumor immune microenvironment, need to be assessed before their use in the clinic [8,215].

Cancer chemotherapy and radiotherapy are intended to eliminate cancer cells largely by generating DNA damage. In a typical situation, the body’s inbuilt DNA damage response machinery will detect and fix any affected DNA. The cells have a chance of survival if the damaged lesions can be fixed. To precisely and effectively kill cancer cells with therapies that generate DNA damage, it is crucial to take advantage of certain defects in the DDR machinery that are present in cancer cells but not in normal cells [216]. By personalizing treatment to patients whose tumors lack specific DDR functions, targeted therapy offers the promise of a larger therapeutic window. The majority of cancers, if not all of them, will have lost at least one or more crucial DDR pathway resulting in an increased dependence on the compensatory pathways that are still functional and capable of removing arising DNA damage and thereby preventing cell death. This feature can be exploited in the design of specific DDR inhibitors that target crucial components of the above-mentioned—non-defective DNA repair pathway that is used by cells for survival. This phenomenon known as synthetic lethality may help to selectively eliminate cancer cells leaving the normal cells unharmed (such cells still have the first pathway intact and can repair the DNA damage) [217,218,219]. This strategy has been successful in the past, as evidenced by the use of PARP inhibitors in the treatment of BRCA-mutated cancers [220,221]. More recently, the expansion of effective and selective agents that target DDR signaling components has emerged as a promising therapeutic option, and this trend is expected to continue [222]. There are also several different examples of DDR-targeted drugs coupled with radiation and chemotherapeutic drugs that have shown evidence of success in preclinical and clinical testing [216,223]. On the other hand, there are a number of crucially important questions such as what dose of DDR inhibitor will serve as an efficient radiosensitizer, how much benefit will be derived from prolonged exposure to the DDR inhibitor following delivery of radiation-induced damage, or even whether radiosensitization by the DDR inhibitor is distinctive in particular DDR-deficient genetic characteristics. To determine whether enhanced antitumor cell effectiveness is paired with increases in healthy tissues toxicity, or if there is evidence that suggests an improved therapeutic index, is another matter of concern when combining different approaches. Although anticancer activity and normal tissue toxicity can be evaluated using distinct models, it is preferable to make use of a syngeneic or orthotopic immune-competent rodent model, in which antitumor activity and normal tissue toxicity can be evaluated concurrently with the effect of such a combination on the antitumor immune response. The conjunction of DDR therapeutics with chemotherapies that induce DNA damage also has its challenges. There are two primary reasons for this: the first is that chemotherapies are administered systemically, and the second is that they have a tendency to have intersecting toxicities as DDR inhibitors, specifically gastrointestinal and bone marrow toxicity. As a consequence of this, a significant number of clinical studies have been stopped since the risks involved are intolerable. This topic was thoroughly reviewed by O’Connor in an excellent paper [224] and the toxicity of DDR inhibitors was discussed recently by Martorana et al. [225]. Moreover, the resistance to DDR inhibitors is a major concern that influences the success of the therapy [226].

Large-scale functional genomic screens have identified a growing number of genetic vulnerabilities throughout the DDR landscape beyond PARPi. This has led to a variety of novel synthetic lethal targets and inhibitors, which may expand DDR inhibitor use beyond HR and address PARPi resistance. For example, berzosertib (VX-970) is a first-in-class ATR inhibitor that has been examined as monotherapy or in combination with cytotoxic chemotherapy drugs (topotecan, carboplatin, cisplatin, and gemcitabine) in phase 1 clinical studies and showed overall safety and clinical benefits in patients. These findings prompted berzosertib–cytotoxic chemotherapy research. For example, berzosertib plus gemcitabine improved PFS in a randomized phase 2 trial of platinum-resistant ovarian cancer. BAY 1895344 constitutes another oral selective and potent ATR inhibitor developed and tested in vivo. Preclinical investigations of this agent as monotherapy in DDR deficit tumors showed promising synergistic anticancer activity with DNA-damage-inducing chemotherapy or radiotherapy. The anticancer activity of BAY 1995344 as a single-agent therapy has been demonstrated to be quite promising; hence, more research is currently being conducted in combination with a PARPi (niraparib) or an immune checkpoint inhibitor (pembrolizumab). The effectiveness and safety of combining PARPi with anti-PD1/PD-L1 antibodies in treating various cancers have been the subject of multiple clinical investigations. There were no unexpected adverse events or additive toxicities seen in the MEDIOLA trial (NCT02734004), a phase 1/2 study examining the combination of olaparib and durvalumab (anti-PD-L1 antibody) in four different types of cancer: germline BRCA-mutated metastatic breast cancer, germline BRCA-mutated platinum-sensitive relapsed ovarian cancer, relapsed gastric cancer, and SCLC. A promising anticancer activity was also revealed in a phase 2 trial of melanoma patients treated with the combination of ATR inhibitor ceralasertib with durvalumab. This topic was extensively covered in other recent reviews [227,228].

The tumor immune microenvironment is profoundly impacted by tumor-promoting inflammation, which in turn affects the success of cancer immunotherapy. Evidence is mounting that inhibiting DDR in conjunction with an immune checkpoint inhibition may increase inflammation in the tumor microenvironment, hence improving immune detection and the killing of cancer cells. Micronucleus and cytosolic chromatin fragments generation are both increased in cancer cells treated with DDR inhibitors due to the DSBs occurrence and genomic instability induction. Pattern recognition receptors (PRRs), which are part of the innate immune system and identify pathogenic nucleic acids such as virus DNA or RNA, recognize the cytosolic DNA produced from DSBs and MNs. The detection of cytosolic DNA by PRRs initiates IFNγ signaling and accompanying innate and adaptive immune responses, similarly as in the case of DAMPs, facilitating the anti-tumor immune response [228]. Several genotoxic treatments, both in the clinic and in animal models, have been found to up-regulate the cGAS-STING pathway and enhance the efficacy of cancer immunotherapy. Cancer immunotherapy can be improved by activating the cGAS-STING pathway, which has been linked to the inhibition of ATM, ATR, CHK1, and PARP [229]. This topic was reviewed by Huang et al. and Wang et al. who summarized recent clinical trials involving combinations of immune checkpoint inhibitors with DDR-directed therapies [228,230]. 

Despite the significant response of melanoma, non-small-cell lung cancer, and lymphoma patients to PD-1/PD-L1-directed treatments, fewer benefits were observed in patients suffering from other cancer types. This could be a result of a scarcity of tumor antigens, poor antigen presentation, and the composition of the tumor microenvironment. The establishment of clinical biomarkers for anti-PD-1/PD-L1 treatments is therefore an urgent need. Most of the research focused on PD-L1 as a prognostic marker of treatment response. However, the inducible nature of PD-L1 expression and non-uniform expression of PD-L1 even in a single tumor adds another layer of complexity to the problem [11,200,231]. Furthermore, this therapeutic approach may be especially useful for a subset of patients who share certain DDR and immune biomarkers (e.g., HR-deficiency [232], TMB-high [233], MSI-high [234,235], deficiency in MMR [236,237,238], and profound expression of immune checkpoint molecules and their variation [239,240]). For example, TMB is associated with the production of abnormal proteins that are degraded proteolytically, leading to the production of neoantigens recognized by T-cells that release IFNs and lead to the stimulation of IRF and PD-L1 up-regulation. Furthermore, both BER and MMR-deficient cells will produce more neoantigens, leading to the up-regulation of PD-L1. Additionally, cells defective in HR and NHEJ pathways may respond better to the anti-PD-L1 therapies due to the release of DAMPs in response to chemotherapy or radiotherapy [241].

Moreover, drug resistance has considerably reduced the clinical benefits of immunotherapy [200]. Among the resistant mechanisms involving the DDR in response to immune checkpoint therapy, several factors can be distinguished including low neoantigen burden, low PD-L1 expression in certain types of tumors, down-regulated tumor MHC expression, recruitment of immunosuppressive cells, and the release of cytokines in the tumor milieu. Nevertheless, by increasing DNA damage with DDR pathway inhibitors, tumor microenvironment inflammation can be altered, immunogenic cancer cell death can be triggered, and anticancer immune responses can be stimulated [242]. For example, inhibiting ATR increases the inflammatory IFN response and cytokine gene expression that is triggered by radiation, both in vivo (especially CCL2, CCL5, and CXCL10) and in vitro (CCL3, CCL5, and CXCL10) [243]. Combining an ATR inhibitor with an immune checkpoint blocker is justified since, in addition to increasing DNA damage, the former also decreases PD-L1 levels and boosts anti-tumor immune responses [244]. 

**Figure 5:**
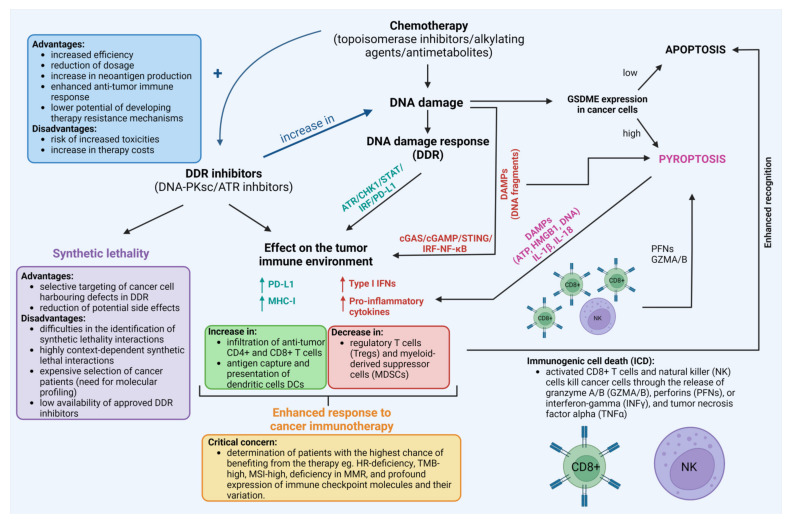
The existing links between the use of chemotherapeutic drugs, DNA damage response (DDR) inhibitors, and immunotherapy with the crucial questions, advantages, and disadvantages of their use. For reference see Section 7. Created with BioRender.com accessed on 1 February 2023.

Furthermore, various in vitro and in vivo studies have shown that classical chemotherapeutic drugs such as platinum compounds including cisplatin [245,246,247,248,249,250] and lobaplatin [251,252,253], antimetabolites including 5-FU [254] and decitabine [255], or topoisomerase inhibitors such as doxorubicin [256,257,258] may trigger cancer cell pyroptosis [259]. It appears that the expression of gasdermines (GSDMs) in target cancer cells determines whether chemotherapeutic agents and cytotoxic lymphocytes promote pyroptosis or apoptosis. This tumor suppressor gene is frequently suppressed in cancer cells, most likely as a result of promoter methylation. Therefore, DNA methyltransferase inhibitors such as decitabine may sensitize cancer cells to pyroptosis by re-establishing gasdermin E (GSDME) expression. By releasing inflammatory chemicals into the extracellular environment, pyroptotic cells trigger an inflammatory and immunological response. Cells undergoing pyroptosis produce molecules that contribute to inflammation and therefore may boost anti-PD-L1 therapies. These inflammatory factors include pro-inflammatory cytokines IL-1β, IL-18, and DAMPs such as ATP, DNA, or HMGB1, and in doing so they regulate the proportions of tumor-infiltrating immune cells such as T cells, NK cells, DCs, monocytes, and MDSCs [260,261,262,263]. Pyroptosis, which is a highly immunogenic form of cell death, generates local inflammation and draws inflammatory cell infiltration. This presents a significant potential to alleviate the immunosuppression of tumor microenvironments and elicit a systemic immune response in the treatment of solid tumors [262]. Indeed, increased levels of GSDME in cancer cells resulted in a substantial rise in the number of antigen-specific CD8+ T cells and intra-tumoral NK cells. It also resulted in a boost in the expression of granzyme B, perforins, IFN-γ, and TNF-α in TILs, as well as an increase in the phagocytosis of tumor cells by tumor-associated macrophages in a melanoma xenograft model treated with serine/threonine-protein kinase B-raf (BRAF) and mitogen-activated protein kinase kinase (MEK) inhibitors [264]. It has been, however, hypothesized that a persistent inflammatory state raises the likelihood of cancer development. Because of the local inflammatory milieu, angiogenesis, invasion, and metastasis are all more likely to occur. It seems that the level of equilibrium that exists in the microenvironment between pro-tumorigenic factors and ones that are anti-tumorigenic is what controls the formation of tumors. Moreover, the induction of pyroptosis may lead to damage to normal tissues. Nevertheless, the precise control of inflammasome activation and pyroptosis could present an opportunity to significantly enhance the efficacy of immune treatment in the near future [262,265,266]. This is evidenced by the number of clinical drugs and pre-clinical compounds that activate pyroptosis in cancer cells and their combinations with immune checkpoint inhibitors as reviewed recently by Wang et al. [263]. The existing links between DDR induced by chemotherapeutic drugs, DDR-inhibitors, and immunotherapy are presented in Figure 5. 

## 8. Conclusions

The understanding of the influence of the combinations of chemotherapeutic drugs and DDR inhibitors on the tumor immune microenvironment may help to increase the effectiveness of cancer treatment and limit the detrimental influence of therapies on normal cells, alleviating potentially life-threatening side effects. Despite the promise it holds for cancer patients, still many concerns remain. One of them is the appropriate selection of patients that could benefit the most from the combinations of drugs. The advances in the comprehension of the molecular biology of cancer and its response to potentially complementary therapies now help to increase the likelihood of therapy success. 

## Data Availability

Not applicable.

## Abbreviations

53BP1	TP53-binding protein 1
5-FU	5-fluorouracil
AKT	RAC-alpha serine/threonine-protein kinase
APC	Antigen-presenting cell
APAF-1	Apoptotic protease-activating factor 1
ATM	Serine-protein kinases ataxia telangiectasia mutated
ATRIP	ATR-interacting protein
BAD	Bcl2-associated agonist of cell death
BAP31	B-cell receptor-associated protein 31
BER	Base excision repair
BRAF	Serine/threonine-protein kinase B-raf
BRCA1/2	Breast cancer type 1 susceptibility protein 1/2
CDC25A/C	M-phase inducer phosphatases
CDK	Cyclin-dependent kinase
cGAS	Cyclic GMP-AMP synthase
CHK1/2	Serine/threonine-protein kinase CHK1/2
CK2	Casein kinase II
COX2	Cyclooxygenase-2
CRT	Calreticulin
CSC	Cancer stem cells
CtIP	CtBP-interacting protein
DAMPS	Damage-associated molecular pattern molecules
DCs	Dendritic cells
DDR	DNA damage response
DISC	Death-inducing signaling complex
DNA-PKcs	DNA-dependent protein kinase catalytic subunit
DSBR	Double-strand break repair
DSBs	Double-strand breaks
ED	Extracellular domain
eIF2α	Eukaryotic translation initiation factor 2A
EMT	Epithelial-to-mesenchymal transition
ERK	Extracellular signal-regulated kinase
ES-SCLC	Extensive-stage small cell lung cancer
EXO1	Exonuclease 1
FA	Fanconi anemia
FADD	FAS-associated death domain protein
FdUMP	5-fluoro-2′-deoxyuridine 5′-monophosphate
FdUTP	Fluorodeoxyuridine triphosphate
FLIP	Flice-like inhibitory protein
FOXO1	Forkhead box protein O1
GADD45	45kd-growth arrest and DNA damage
GDSM	Gasdermin
GZMA/B	Granzyme A/B
HIF-1α	Hypoxia-inducible factor alpha
HMG1/2	Nonhistone chromosomal high-mobility group 1 and 2
HMGB1	High mobility group protein 1
HR	Homologous recombination
hUBF	Highly similar to Human upstream binding factor
ICD	Immunogenic cell death
ICLs	Interstrand crosslinks
ICT	Intracellular cytoplasmic tail
IFN	Interferon
IL	Interleukin
ILC2s	Group 2 innate lymphoid cells
irAEs	Immune-related adverse effects
IRF	Interferon regulatory factor
ITIM	Immunoreceptor tyrosine-based inhibitory motif
ITSM	Immunoreceptor tyrosine-based switch motif
JAK	Tyrosine-protein kinase
JNK	Jun amino-terminal kinase
KU70/80	X-ray repair cross-complementing protein 5/6
M2	Tumor-associated macrophages M2
mAbs	Monoclonal antibodies
MCM2/7	Minichromosome maintenance complex
MDC-1	Mediator of DNA damage checkpoint protein 1
MDM2	Mouse double minute 2 homolog
MDSC	Myeloid-derived suppressor cells
MDSCs	Myeloid-derived suppressor cells
MEK	Dual specificity mitogen-activated protein kinase kinase
MHC	Major histocompatibility complex
MLH1	DNA mismatch repair protein MLH1
MMR	Mismatch repair
mPGES1	Prostaglandin E synthase
MRE11	Double-strand break repair protein MRE11
MSH2/6	DNA mismatch repair protein MSH2/6
MSI	Microsatellite instability
mtDNA	Mitochondrial DNA
mTOR	Serine/threonine-protein kinase mTOR
Nal-IRI	Liposomal irinotecan
NBS1	Nibrin
nDNA	Nuclear DNA
NER	Nucleotide excision repair
NF-Kβ	Nuclear factor NF-kappa-B p105 subunit
NHEJ	Non-homologous end joining
NK	Natural killer cells
OMI	Serine protease HTRA2
OS	Overall survival
P2RX7	P2X purinoceptor 7
PAR	Polymer ADP-ribose residues
PARP-1	Poly [ADP-ribose] polymerase 1
PARPi	PARP inhibitor
PD-1/PD-L1	Programmed death receptor-1/programmed death ligand-1
PERK	Eukaryotic translation initiation factor 2-alpha kinase 3
PFN	Perforins
PFS	Progression-free survival
PGE2	Prostaglandin E2
PI3K	Phosphatidylinositol-4,5-bisphosphate 3-kinase
PKC	Protein kinase C
PLCg-1	1-phosphatidylinositol 4,5-bisphosphate phosphodiesterase gamma-1
PLK	Serine/threonine-protein kinase PLK
PRR	Pattern recognition receptors
PS	Phosphatidylserine
PTEN	Phosphatidylinositol 3,4,5-trisphosphate 3-phosphatase and dual-specificity protein phosphatase
RAD50	DNA repair protein RAD50
RAD51	DNA repair protein RAD51 homolog 1
RAD52	DNA repair protein RAD52 homolog
RAP80	Receptor-associated protein 80
RNF4, -8, -168	E3 ubiquitin-protein ligases
ROS	Reactive oxygen species
RPA	Replication protein A
SCLC	Small-cell lung cancer
SHP1/2	Src homology region 2 domain containing phosphatases
SMACK	Diablo IAP-binding mitochondrial protein
SSBR	Single-strand break repair
ssDNA	Single-stranded DNA
STAT	Signal transducer and activator of transcription
STING	Stimulator of interferon genes protein
TBP	TATA binding protein
TCR	T-cell receptor
TGF-β	Transforming growth factor β
TICs	Tumor-initiating cells
TILs	Tumor infiltrating lymphocytes
TLR	Toll-like receptor
TLS	Translesion DNA synthesis
TM	Transmembrane domain
TMB	Tumor mutational burden
TNF-α	Tumor necrosis factor α
TOPBP1	Topoisomerase 2-binding protein 1
TP53	Cellular tumor antigen p53
Tregs	Regulatory T cells
WAF1/CIP1	Cyclin-dependent kinase inhibitor 1
XLF	Non-homologous end-joining factor 1
XRCC4	X-ray repair cross-complementing protein 4
